# In vivo effects of antibodies from patients with anti-NMDA receptor encephalitis: further evidence of synaptic glutamatergic dysfunction

**DOI:** 10.1186/1750-1172-5-31

**Published:** 2010-11-26

**Authors:** Mario Manto, Josep Dalmau, Adrien Didelot, Véronique Rogemond, Jérôme Honnorat

**Affiliations:** 1 FNRS - Laboratoire de Neurologie Expérimentale, ULB, Belgium; 2 Department of Neurology, Division of Neuro-oncology, University of Pennsylvania, 3400 Spruce Street, Philadelphia, USA; 3 Hospices Civils de Lyon, Hôpital Neurologique, Centre de Référence Maladie Rare "Syndromes neurologiques Paranéoplasiques", Neurologie B, F-69677 Bron, France; 4 INSERM, U842, Lyon, F-69372 France; Université de Lyon, Lyon1, UMR-S842 Lyon, F-69003 France

## Abstract

**Background:**

A severe encephalitis that associates with auto-antibodies to the NR1 subunit of the NMDA receptor (NMDA-R) was recently reported. Patients' antibodies cause a decrease of the density of NMDA-R and synaptic mediated currents, but the *in vivo *effects on the extracellular glutamate and glutamatergic transmission are unknown.

**Methods:**

We investigated the acute metabolic effects of patients' CSF and purified IgG injected in vivo. Injections were performed in CA1 area of Ammon's horn and in premotor cortex in rats.

**Results:**

Patient's CSF increased the concentrations of glutamate in the extracellular space. The increase was dose-dependent and was dramatic with purified IgG. Patients' CSF impaired both the NMDA- and the AMPA-mediated synaptic regulation of glutamate, and did not affect the glial transport of glutamate. Blockade of GABA-A receptors was associated with a marked elevation of extra-cellular levels of glutamate following a pretreatment with patients' CSF.

**Conclusion:**

These results support a direct role of NMDA-R antibodies upon altering glutamatergic transmission. Furthermore, we provide additional evidence in vivo that NMDA-R antibodies deregulate the glutamatergic pathways and that the encephalitis associated with these antibodies is an auto-immune synaptic disorder.

## Introduction

Antibodies to the N-methyl-D-aspartate (NMDA) subtype of glutamate receptor have been identified in a newly-described encephalopathy [1]. One of the antigens corresponds to extracellular epitopes of NR1 subunit of the NMDA receptor (NMDA-R). Typically, patients are young women with teratoma of the ovary and presenting with acute psychiatric manifestations, seizures, dyskinesias, hypoventilation and autonomic instability [2]. Early removal of the teratoma followed by plasma exchange, intravenous immunoglobulins, and corticosteroids administration frequently results in neurological improvement and even full recovery [3].

Recent studies showed that patients' antibodies cause a selective and reversible decrease in NMDA-R surface density and synaptic localization that correlates with antibody titers. The mechanism of this decrease is selective antibody-mediated crosslinking and internalization of the receptors. Furthermore, whole-cell patch clamp recordings of miniature excitatory postsynaptic currents in cultured rat hippocampal neurons showed that patients' antibodies specifically decreased synaptic NMDA-R-mediated currents. In contrast, patients' antibodies did not alter the localization or expression of other glutamate receptors or synaptic proteins, number of synapses, dendritic spines, dendritic complexity, or cell survival. NMDA-R cluster density was also dramatically reduced in the hippocampus of rats infused with patients' antibodies, similarly to the decrease of NMDA-R immunostaining observed in the hippocampus of autopsied patients [4].

Although patients' antibodies cause a dramatic reduction of NMDA-R in vivo, the metabolic effects on the regulation of glutamate are unknown. An alteration of the regulation of glutamate would further support the role of NMDA-R Ab in the pathogenesis of the disorder, given the crucial functions of glutamate in these regions. To test this hypothesis, we conducted experiments in vivo using microdialysis and determined whether patients' CSF antibodies alter the extra-cellular concentrations of glutamate. We evaluated the effects of NMDA-R Ab on the NMDA- and AMPA (alpha-amino-3-hydroxy-5-methyl-4-isoxazole propionic acid)-mediated regulation of glutamate. We also investigated the potential effects of NMDA-R Ab on the glial transport of glutamate. Moreover, we used bicuculline, an antagonist of GABA-A receptors, in order to unravel a susceptibility to the blockade of GABA-A receptors following a pretreatment with NMDA-R Ab. In addition, we studied the effects of infusion of GABA (gamma-amino-butyric acid) after blockade of the alpha2-delta subunit of voltage-gated calcium channels (VGCC) with pregabalin, to assess the responsiveness of the glutamatergic synapses to exogeneous GABA when the presynaptic release of glutamate was blocked. Finally, we studied the effects of NMDA-R Ab on nitric oxide (NO), given the intimate link between the NMDA pathway and NO in the brain.

## Methods

### Cerebrospinal fluid and IgG purification

All samples were dialyzed against phosphate buffered saline, and solutions were used at pH of 7.3. All the CSF used in the present study had pH and glucose levels within the normal range.

#### Patients' CSF positive for NMDA-R Ab and purified IgGs

Cerebrospinal fluid (CSF) was obtained from 6 patients with encephalitis (4 from University of Pennsylvania-USA and 2 from University of Lyon-France. These last 2 CSF have the reference 9049 and 9052, see later in the text) associated with antibodies to NR1/NR2 heteromers of the NMDA receptor. These CSF samples are referred as *patients' CSF*. In all cases the CSF was collected at symptom presentation, before any treatment. In addition, we also used purified IgGs in experiments to confirm the results found with patients' CSF. Purified IgGs were obtained from the serum of one patient with NMDA-R-Ab (patient 9052). IgGs were adsorbed to protein A-Sepharose beads (protein A Sepharose 4 fast flow; Amersham Biosciences, Saclay, France) and eluted with sodium citrate (0.5 M, pH 2.5). After neutralization, samples were dialyzed overnight at 4°C against Ringer solution (Fresenius Kabi, Sèvres, France) and sterilized by filtration with 0.22 m filters as previously described [5]. The presence of NR1/NR2 antibodies was demonstrated in all patients as reported earlier[2].

#### Controls' CSF and purified IgGs

Controls' CSF (n = 5) were obtained from 2 patients with herpes simplex encephalitis (HSE), 2 patients with neurodegenerative disorders (ND), one with paraneoplastic sensory neuropathy associated with anti-Hu antibodies and a small cell lung carcinoma (sample 9093), and purified IgGs fractions from one patient with cerebellar ataxia and anti-Yo antibodies (see above for the purification method).

### Infusion of Ab and microdialysis

Experiments were approved by the Animal Care Committee of ULB. We made all efforts to reduce animal suffering as much as possible and to reduce the number of animals used for the study. Males wistar rats (weight: 240-430 gr) were anaesthetized with chloral hydrate (400 mg/kg administered ip) prior surgery. *Numbers of rats used for each experiment are indicated in the figure legends.*

Part of the methodology has been reported elsewhere [5]. Briefly, microdialysis guides (CMA12, CMA, Sweden) were inserted in the superior limit of CA1 area and in the premotor area rFr2 according to the atlas of Paxinos-Watson (see also table 1 the respective sites of injection for the experiments carried out and the working hypothesis for each experiment). Coordinates for CA1 area and rFr2 were (related to bregma): A/P -5.2 mm, Lat 4.5 mm, D/V -2.4 mm, and A/P + 2 mm, Lat 1 mm, D/V -1 mm, respectively. Dental cement was used to fix the guides on the skull. Rats were anaesthetized with chloral hydrate (400 mg/kg ip) [5]. We selected this procedure of continuous anaesthesia because (a) baseline measurements are more stable due to absence of interference of voluntary motor activity on neurotransmission, and (b) we discovered that in vivo glutamate measurements are highly sensitive to mechanical perturbations that might occur in the environment when rats hit unexpectedly the sides of the cage or any structures around. A needle (Hamilton point style 4, Hamilton) was inserted in the guide. The extremity of the needle was located between -2.4 and -4.4 mm for CA1 zone, and between -1 and -2 mm for rFr2 area. Infusion of antibodies was performed using a micropump (CMA100, CMA, Sweden). Rats were lying over a temperature regulator (Heating Controller 872/1, Harvard apparatus). Indeed, it has been shown that the temperature is a key-factor for NMDA assessments (the channel kinetics play an important role in determining amplitude and time course of NMDA receptor-mediated postsynaptic currents) [6]. Microdialysates were collected every 10 minutes (unless specified). Ringer's solution (composed of NaCl 148 mM, CaCl2 1.1 mM, KCl 4 mM; optimized at pH 7.2 with NaHCO3 10 mM) was used.

**Table 1:** Sites of injection for the experiments carried out

Experiment	Site of injection	Working hypothesis
Dose-response study^a,b,c^	CA1 rFr2	NMDA-R Ab impair the glutamate concentrations in the extra-cellular space

NMDA and AMPA regulation^a,b,c,d^	CA1	NMDA-R Ab impair the NMDA- and AMPA-mediated regulation of glutamate

Inhibition of glial transport^a,b^	CA1	NMDA-R Ab impair the glial transport of glutamate

Blockade of GABA-A receptors^a,b^	CA1	Bicuculline enhances the concentrations of glutamate in case of pre-treatment with NMDA-R Ab

Infusion of GABA^c^	rFr2	Infusion of GABA decreases the concentrations of glutamate after administration of pregabalin

Effects on concentrations of nitric oxide (NO)^a,b^	rFr2	NMDA-R Ab increase the concentrations of NO

^a^Use of controls' CSF; ^b^Use of patients' CSF; ^c^Use of purified IgG from a patient positive for NMDA-R antibodies; ^d^Use of purified IgGs from a patient positive for anti-Yo antibodies.

The injection procedure itself did not affect the values of metabolites collected by microdialysis. This was demonstrated by the following experiment (microdialysates collected every 10 min: 3 basal measurements followed by injection of Ringer solution 5 μL at a flow rate of 1 μL/min at + 30 min, followed by 3 measurements post-injection). In 4 rats, the baseline values (±SD) of extra-cellular glutamate in CA1 zone were: 2.13 ± 0.30 μM at time 10 min, 2.05 ± 0.24 at time + 20 min, 1.95 ± 0.35 μM at time + 30 min, 2.23 ± 0.33 μM at time + 45 min, 2.13 ± 0.49 μM at time + 55 min, 1.98 ± 0.43 μM at time + 65 min (analysis of variance: F = 0.279, p = 0.917).

### Analysis of metabolites

Concentrations of glutamate were determined using a CMA600 device (CMA, Sweden). Linearity regression coefficient (R^2^) determined locally is 0.9984 [7]. The following experiments were conducted:

#### -Dose-response study

We first determined whether controls' CSF versus patients' CSF modified the extra-cellular concentrations of glutamate. Following the observation of an increase in glutamate concentrations with patients' CSF, we studied the dose response effect of one patient's CSF (9049) using 2 dilutions (half-dose and 1/1). Glutamate concentrations were determined at baseline and 30 minutes following infusion of each dilution. An equilibration period was used to allow stable baseline measurements. We confirmed the dose response effect using purified IgGs at 4 dilutions (1/8, 1/4, 1/2 and 1/1). The volume injected was 5 μL, with a flow rate of 1 μL/min.

#### - NMDA and AMPA regulation

The NMDA effect [5,7] was evaluated using the following procedure: we first determined the concentrations of glutamate in 4 successive samples starting 60 minutes after infusion of a control solution (CSF from neurological patients without NMDA-R Ab) or patients' CSF (volume of 5 μL; flow rate of 1 μL/min for 5 min). We subsequently infused NMDA by reverse dialysis (20 mM dissolved in Ringer solution, infusion flow 1 μL/min) and measured glutamate in the 4 consecutive samples. The effect of 2-amino-5-phosphonovaleric acid (APV, a selective NMDA receptor blocker; 50 μM by reverse dialysis) was evaluated 30 minutes after infusion of a control solution or patients' CSF. To assess the interaction with the AMPA pathway, AMPA and the AMPA blocker DNQX (6,7-dinitro-quinoxaline-2,3-dione) were used at doses of 5 mM and 500 μM, respectively [7-9]. AMPA and DNQX were administered 1 hour after the infusion of the control solution or the patients' CSF. AMPA was administered alone and in combination with NMDA. DNQX was administered either alone or in combination with NMDA 20 mM.

#### -Inhibition of glial transport

Since astrocytes play a major role in the removal of glutamate from the extracellular compartment [10], the effects of the glutamate transport inhibitor L-2,4-trans-pyrrolidine-dicarboxylate (PDC; 10 mM) were studied to evaluate the consequences of glial transport inhibition on the concentrations of glutamate in the extra-cellular space in presence of NMDA-R Ab. PDC partly mimics reverse glutamate uptake [11]. PDC was infused after 60 minutes by reverse dialysis, either following the injection of the control CSF 9093 or following the injection of patients' CSF (CSF 9049). The infusion of PDC lasted 10 minutes. We determined the ratios of increase of glutamate concentrations induced by PDC (glutamate concentrations post-PDC administration divided by glutamate concentrations pre-PDC administration).

#### -Blockade of GABA-A receptors and infusion of GABA

In order to study the effects of blockade of GABA-A receptors, we infused bicuculline by reverse dialysis in loco at a concentration of 20 μM during 30 minutes, after infusion of the control CSF 9093 or the NMDA-R Ab positive CSF 9049. We determined the concentrations of glutamate before and after blockade of GABA-A receptors. We also analyzed the effects of infusion of gamma-aminobutyric acid (GABA; 50 μM) following pre-administration of pregabalin (a selective blocker of VGCC) to estimate the rate of sensitivity to GABA when presynaptic release of glutamate was blocked. This set of experiment was carried out following the infusion of purified IgGs (positive for NMDA-R antibodies).

#### Measurement of nitric oxide (NO)

NO was measured using a selective microsensor (Iso-NOPF100; Apollo 1000, World Precision Instruments) inserted near the tip of the cannula at the site of infusion in the brain. Calibration was performed using S-nitroso-Nacetyl-D,L-penicillamine (SNAP, Sigma) as reported by Alvarez et al. [12]. We administered 7-nitroindazole (7-NI), a selective blocker of nNOS, in order to test the hypothesis that changes in NO concentrations were of neuronal origin. 7-NI was infused in loco during 5 minutes (dose: 200 μM). NO was measured at baseline, following infusion of a control solution or CSF with NMDA-R Ab (time T50 to T60), and following administration of 7-NI (time T60 to T70).

#### Evaluation of microperfusion

We also assessed the microperfusion in the sites of injection (CA1, rFr2) using laser Doppler flowmetry (LDF), in order to confirm that the microperfusion was preserved during the experiments. A laser flow probe was inserted near the tip of the microdialysis guide in order to monitor the blood flow locally in the brain (Oxylab, Oxford optronix microvascular perfusion monitor). This technique allows the early detection of bleeding (immediate drop in blood flow). The technique has been first validated in 12 rats. We determined the blood flow (expressed in arbitrary units BPU - blood per units - allowing evaluation of *relative *changes in perfusion at the beginning and at the end of each experiment [13]. Rats with impaired blood flow were excluded from the analysis (3 rats with regional blood flow decreasing below 45% of basal values, whereas the regional blood flow values remained above 65% of baseline values in all the other rats for all the experiments performed).

#### Histological verification

**Figure 1:**
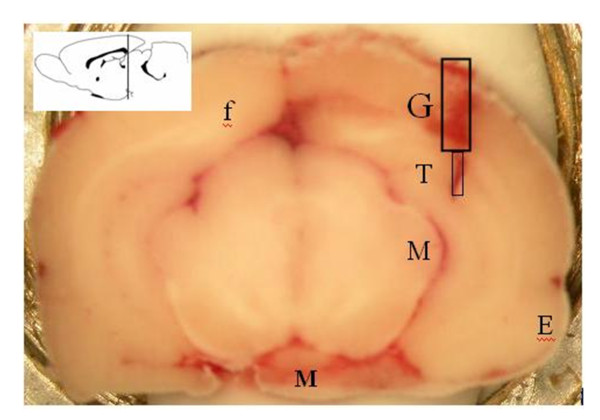
**Example of histological section at the level of CA1 area after experiment**. Location of the guide (G) and tip (T) of the microdialysis probe in right CA1 of the rat after experiment. Ent: entorhinal cortex; MG: medial geniculate nuclei; MP: medial mammillary nucleus; fmj: forceps major corpus callosum.

We assessed the localization of the injection site for each rat on frozen brain sections in a way very similar to a method previously published [14]. Only data with correct probe location (the exposed dialysis membrane located in the target region) were analysed (3 rats excluded). Figure 1 illustrates an example of histological section at the level of CA1 area.

#### Statistical analysis

Data were exported to Microsoft Excel. Statistical analysis was performed using Sigma Stat (Jandel Scientific, Germany). The normality of data was assessed with the Kolmogorov-Smirnov test, prior selection of parametric versus non parametric procedures. We applied the Mann-Whitney rank sum test to compare the concentrations of glutamate at baseline (before infusion of CSF) in the 2 groups (control group versus NMDA-R Ab-positive group) and to compare the effects of infusion of CSF in the control group and in the NMDA-R Ab-positive group. For the dose-response study, a linear regression was computed with 95% confidence and prediction intervals for the NMDA-R Ab-positive group. To analyse the effects of AMPA, NMDA, APV, AMPA and DNQX on the concentrations of glutamate, we applied the analysis of variance on ranks, followed by the Tukey test. To compare the effects of trans-PDC in the 2 groups of rats (rats infused with control solution versus rats infused with NMDA-R Ab), we used the Student test. The effects of blockade of GABA-A receptors on extra-cellular concentrations of glutamate were assessed with the analysis of variance followed by the Bonferroni test. A similar procedure was applied to assess the effects of infusion of GABA after administration of pregabalin. We used the analysis of variance followed by the Tukey test to evaluate the effects of 7-NI on NO after the infusion of control solution or NMDA-R Ab.

## Results

### 1. Patients' CSF increase glutamate concentration in the extra-cellular space in a dose-dependent manner

**Figure 2:**
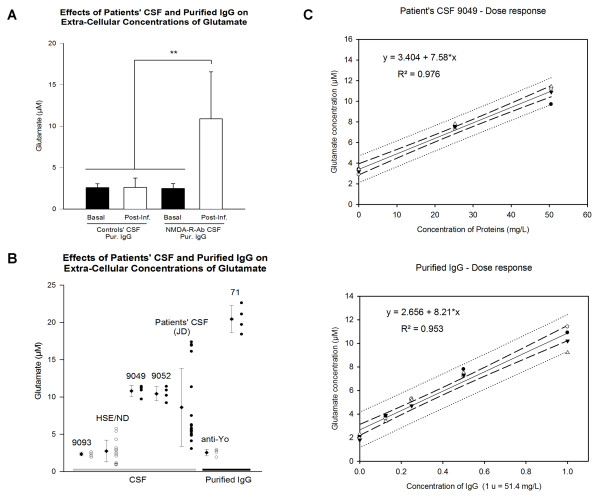
**Effects on extra-cellular concentrations of glutamate**. A: comparison of the glutamate concentrations measured before (basal) and after infusion of CSF or purified IgGs of patients (Post-Inf.). Each sample (5 control CSF, 6 patients' CSF with NMDA-R-Ab, one purified IgGs positive for NMDA-R-Ab and one purified IgGs positive for anti-Yo antibodies) was infused in 4 different rats. Means and SDs are represented. Measurements were made at baseline (before infusion), and between 30 and 60 minutes after CSF or purified IgGs infusion. **B**: point plot (one point corresponds to one rat injected) of the extra-cellular concentrations of glutamate following infusion of CSF (open circles: controls' CSF; filled circles: patients' CSF), and purified IgGs (open circles: control IgGs from a patient with Yo-Ab; filled circles: purified IgGs from a patient positive for NMDA-R-Ab) measured in CA1 zone between 30 and 60 minutes after CSF infusion. **C**: Top panel: concentrations of extra-cellular glutamate according to the dilution of patients' CSF infused in the CA1 area. Four rats were infused with 3 successive doses from one patient's CSF positive for NMDA-R-Ab (CSF 9049). Linear regression, 95% confidence intervals (dashed lines) and 95% prediction intervals (dotted lines) are shown. Bottom panel: Similar results were obtained with purified IgG of CSF 9052. Four rats were individually infused with 4 successive doses. **: p < 0.01.

Figure 2A illustrates the concentrations of glutamate measured before and after infusion of controls' CSF (5 controls' CSF), patients' CSF with NMDA-R-Ab (6 patients' CSF) and purified IgGs from one patient with NMDA-R-Ab and as control, one patient with Yo-Ab, respectively. At baseline (before infusion of CSF), concentrations of glutamate were similar in the control group and the patients' CSF group (mean ± SD: control group: 2.59 ± 0.45 μM, patients' CSF group: 2.48 ± 0.60 μM; inter-group difference: p = 0.24). In the control group, infusion of CSF did not change the concentrations of glutamate measured after infusion as compared to baseline values before infusion (mean values ± SD after infusion: 2.61 ± 1.13 μM; infusion effect: p = 0.60). By contrast, infusion of patients' CSF with NMDA-R-Ab and purified IgGs raised significantly the concentrations of glutamate (mean values ± SD after infusion = 10.87 ± 5.69 μM; infusion effect: p < 0.001). Figure 2B shows the concentrations of glutamate after infusion for the subgroups of rats infused with the various CSF (controls' CSF and patients' CSF). In addition, the concentrations of glutamate obtained with the purified IgGs are also shown. Extra-cellular concentrations of glutamate following infusion of control purified IgGs remained unchanged (range: 1.91 to 2.79 μM). By contrast, values of glutamate concentrations following infusion of purified IgGs from a patient positive for NMDA-R antibodies were extremely high, ranging from 18.4 to 22.6 μM.

Using patient's CSF 9049 infused in rFr2, or purified IgGs infused in CA1, a dose response effect was identified with a linear relationship (Figure 2C; R^2 ^= 0.976 and 0.953, respectively) between the dilution of patient' CSF and the concentrations of glutamate in the extra-cellular space (p < 0.001).

### 2. Patients' CSF impair NMDA and AMPA-mediated regulation of glutamate

**Figure 3:**
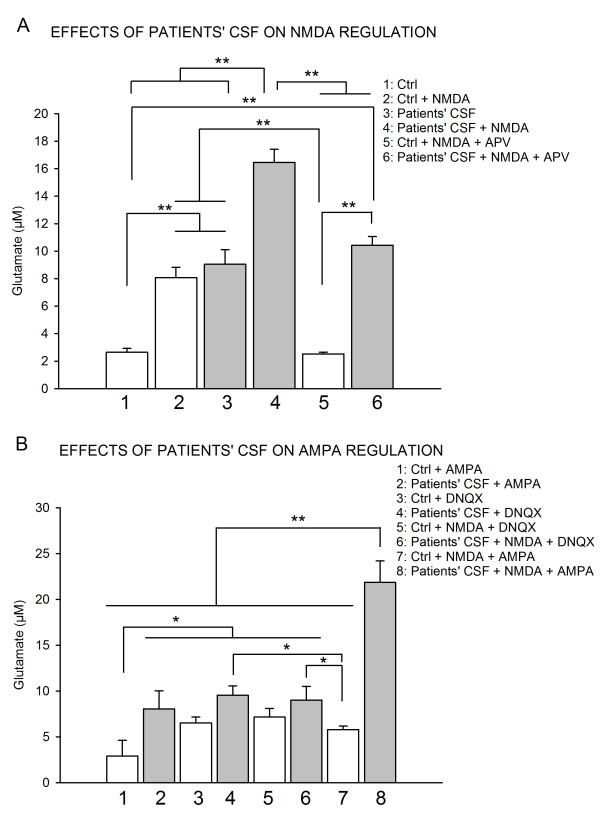
**Effects of patients' CSF on glutamate receptor functions**. **A**: Effects of infusion of patients' CSF on the NMDA-mediated regulation of glutamate. Groups 1 and 2: n = 8 rats injected with 5 control CSF. Groups 3 and 4: n = 8 rats injected with CSF from 5 patients positive for NMDA-R-Ab. Groups 5 and 6: 4 rats injected with a control CSF and 4 rats injected with one CSF positive for NMDA-R-Ab, respectively. Values are mean ± SEM. **B**: Effects of AMPA, AMPA blockade (DNQX), combination of NMDA + DNQX and combination of NMDA + AMPA following administration of NMDA-R-Ab. Groups 1, 3 and 5: 4 rats injected with 4 control CSF. Groups 2, 4 and 6: 4 rats injected with CSF from 4 patients with NMDA-R-Ab. Group 7: 4 rats injected with 1 control CSF. Group 8: 4 rats injected with 1 CSF with NMDA-R-Ab. Values are mean ± SEM. *: p < 0.05; **: p < 0.01

Administration of NMDA increased the concentrations of glutamate in case of pre-infusion with control CSF (from 2.64 ± 0.29 to 8.06 ± 0.76 μM; p < 0.01; Figure 3A) and the basal extra-cellular concentration of glutamate was restored by administration of APV, an antagonist of NMDA-R (2.52 ± 0.12 μM; p = 0.56). NMDA also increased the extra-cellular concentration of glutamate after pre-infusion with patients' CSF (from 9.04 ± 1.05 to 16.45 ± 0.95 μM; p < 0.001) and the glutamate concentration decreased also by subsequent administration of APV (10.42 ± 0.63 μM; p < 0.01). However the concentrations of glutamate were not completely restored by APV, suggesting that NMDA-R-Ab impaired the glutamatergic transmission also via other receptors than NMDA-R. Therefore, in order to assess the AMPA pathway we studied the effect of the administration of AMPA and DNQX (an antagonist of AMPA receptor). Following infusion of AMPA, extra-cellular levels of glutamate were 2.91 ± 1.71 μM in controls and 8.06 ± 1.97 μM in the group NMDA-R antibodies (inter-group difference: p < 0.05; Figure 3B). Interestingly, DNQX had a different effect in rats pre-infused with controls' CSF or patients' CSF. Indeed, when DNQX was administered 1 hour after controls' CSF, glutamate concentration increased from 2.64 ± 0.29 to 6.5 ± 0.66 μM. By contrast, no effect was observed when DNQX was administered after patient's CSF (9.04 ± 1.05 versus 9.53 ± 1.01 μM) suggesting that AMPA receptors were deregulated by the presence of patients' CSF. NMDA had no effect in presence of DNQX on glutamate concentration both in control and patient's CSF groups. However, the effect of concomitant administration of NMDA and AMPA was totally different in the two groups. In the rats infused with patients' CSF, a major increase in the concentrations of glutamate was observed as compared to rats infused with NMDA alone (21.85 ± 2.33 versus 16.45 ± 0.95 μM respectively: p < 0.01). Concomitant administration of AMPA and NMDA decreased the extra-cellular concentration of glutamate in the control group (8.06 ± 0.76 versus 5.8 ± 0.37 μM: p < 0.05). Taken together, these results indicate that patients' CSF modified the balance between AMPA and NMDA receptors. This is consistent with studies on AMPA/NMDA ratios indicating that the balance between these two pathways is required for maintaining physiological activities in neuronal networks [15].

### 3. Patients' CSF have no effect on glial transport of glutamate

We used the glutamate transport inhibitor L-2,4-trans-pyrrolidine-dicarboxylate (PDC) [11] to evaluate the possible role of astrocytes on the increase of glutamate concentrations after infusion of patient's CSF. The percentage of increase of glutamate concentration induced by the inhibition of the glial transport of glutamate was similar in rats infused with control solution (control CSF 9093; increase to 153.8 ± 14.7% as compared to pre-administration of CSF; n = 6 sides) and in rats infused with patients' CSF (CSF 9049; 156.5 ± 19.7%; n = 6 sides; inter-group difference: Student test: p = 0,791).

### 4. Blockade of GABA-A receptors increases the concentrations of glutamate and infusion of GABA reduces the levels of glutamate after blockade of VGCC

**Figure 4:**
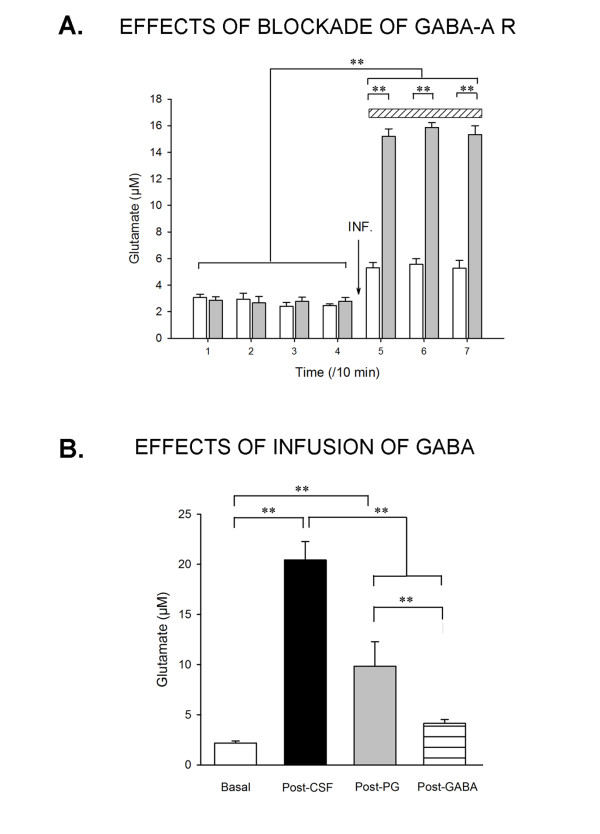
**Interaction with GABA-A receptors and infusion of GABA**. A: Effects of blockade of GABA-A receptors on glutamate levels following infusion of patients' CSF. In rats infused (INF) with CSF with NMDA-R-Ab (9049; n = 3 rats; grey bars), administration of bicuculline (represented by a hatched rectangle) increased markedly the concentrations of glutamate. The bicuculline-induced increase of glutamate was lower in rats injected with the control solution 9093 (n = 3 rats; white bars). **B**: Effects of successive administration of pregabalin (PG) and GABA after infusion of purified IgGs of a patient with NMDA-R-Ab (n = 4 rats). Concentrations of glutamate raised markedly with purified IgGs. Basal: T0 min; Post-NMDA-R-Ab (Post-CSF): post-injection at T30 min; Post-PGB: T90 min post-infusion; Post-Gaba: Post-infusion of Gaba 50 μM at T120 min. Values are mean ± SD. **: p < 0.01

Infusion of bicuculline, an antagonist of GABA-A receptors, increased the levels of glutamate both in case of infusion of control CSF and following infusion of patients' CSF 9049 (Figure 4A; p < 0.001). However, the increase in the group administered with patients' CSF was significantly higher as compared to the increase in the control group, indicating a vulnerability to blockade of GABA-A receptors (group by time interaction: p < 0.001).

For the study on the effects of infusion of GABA, we first confirmed that extra-cellular concentrations of glutamate raised dramatically following infusion of purified IgGs (NMDA-R Ab effect: p < 0.001; Figure 4B). We found that subsequent administration of pregabalin decreased significantly the extra-cellular concentration of glutamate from 20.45 ± 1.81 μM to 9.83 ± 2,48 μM (drop of about 52%; pregabalin effect: p < 0.01). Post-infusion of GABA 50 μM reduced the concentrations of glutamate from 9.83 ± 2.48 μM to 4.1 ± 0.37 μM (drop of about 28% as compared to the concentration obtained after infusion of purified IgGs; GABA effect: p < 0.05). These data showed that GABA still reduced the levels of glutamate even after blockade of pre-synaptic alpha-2-delta subunit of VGCC.

### 4. Patients' CSF increase NO concentrations

**Figure 5:**
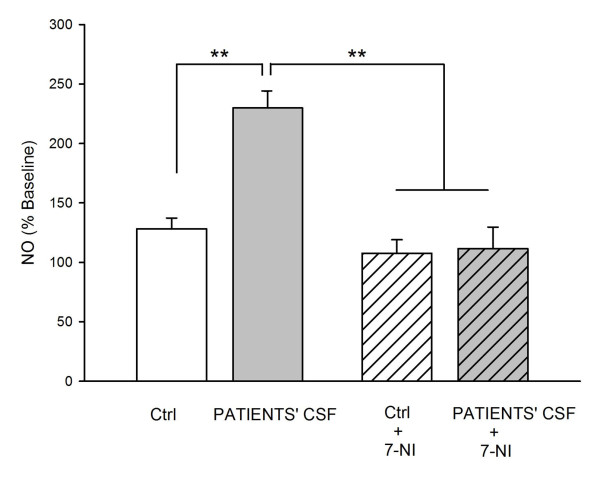
**Effects of patient's CSF on concentrations of nitric oxide (NO)**. Inhibition of nNOS with 7-nitroindazole (7-NI) counteracts the effects of NMDA-R Ab. N = 4 rats infused with control CSF 9093 and n = 4 rats infused with a CSF with NMDA-R-Ab (9049). Values are mean ± SD and are expressed as percentages of baseline measurements. **: p < 0.01

Patients' CSF raised the concentrations of NO as compared to the control solution (p < 0.01; Figure 5). Administration of 7-NI reduced significantly the levels of NO in rats infused with patients' CSF (p < 0.01), arguing for a neuronal origin for the raise in NO concentrations.

## Discussion

The main finding of this study is that patients' CSF alter the extracellular levels of glutamate, suggesting an impairment of the glutamatergic transmission and inducing a susceptibility to AMPA infusion. These results suggest that by decreasing the levels or blocking the extracellular epitopes of the NR1 subunit of the NMDA-R, patients' antibodies induce a hyperglutamatergic state in the brain with an imbalance between NMDA and AMPA pathways. This is the first in vivo demonstration that an antibody targeting NMDA receptors puts the brain circuitry in an AMPA-dependent hyperglutamatergic state. Previous studies have shown that extra-cellular levels of glutamate increase in presence of antibodies targeting glutamic acid decarboxylase (GAD enzyme catalyzing the conversion of glutamic acid into GABA) [5]. Therefore, the increase of extra-cellular levels of glutamate is not specific of NMDA-R antibodies.

### Effects on extra-cellular glutamate

In presence of patients' CSF and purified IgGs, very high concentrations of glutamate were found in the extra-cellular space during NMDA infusion. This is consistent with a dysfunction of the NMDA-related glutamatergic turn-over or an impaired turn-over of receptors, leading to NMDA-related excitotoxicity [5,8,16-18]. Concomitant administration of NMDA and AMPA induced a rise in the extra-cellular concentrations of glutamate up to toxic levels. It is established that high concentrations of glutamate impair the excitability of neuronal networks. The delayed excitotoxic neuronal dysfunction or neuronal death after exposure to high glutamate concentrations appears to play an important role in several neurological disorders [18,19]. Given the key-roles of NMDA receptors in mediating excitatory transmission, an excitotoxic cascade is likely to be triggered at the concentrations found in the present study. In vulnerable neurons, excitotoxic insult induces a sustained positive feedback loop between NMDA-R-dependent current and depolarization-mediated glutamate release, which drives Ca++ elevation and delayed excitotoxicity. We suggest that the balance between AMPA receptors and NMDA receptors might be a key-element for the regulation of glutamatergic neurotransmission in vivo. This is consistent with recent studies on AMPA/NMDA ratios [15]. Fast glutamatergic signalling might be at a compensatory stage or trafficking might be altered by NMDA-R Ab. Indeed, it has been demonstrated that blockade of NMDA-R might stop the AMPA-R endocytosis [20]. However, it should be kept in mind that the NMDA-R-Ab have been shown to cause a reversible reduction of the post-synaptic NMDA-R density as well as NMDA-R-mediated currents in vitro, so that high levels of glutamate may not result in cell death, as suggested by the relative preservation of neurons in autopsy studies and by the reversibility of brain atrophy in severe cases who survived [21]. It is possible that the levels of glutamate in human brain in presence of NMDA-R-Ab do not reach the synaptic threshold necessary to induce a genuine neurodegeneration through the overactivation of AMPA receptors [17].

Although molecular and electrophysiological studies showed that patients' NMDA-R-Ab did not alter the density of synaptic AMPA receptors and AMPA receptor mediated currents in vitro and after infusion of antibodies into the hippocampus of rodents [4], data from the current study suggests that networks of neurons and inhibitory interneurons might be required to observe the imbalance between NMDA and AMPA pathways. Moreover, the current data likely reflects the fact that AMPA receptors are very mobile in the cytoplasmic membrane and microdialysis assesses also extra-synaptic compartments of glutamatergic synapses. About 50% of synaptic AMPA receptors are exchanged with extra-synaptic AMPA receptors in a few minutes [22].

Findings from this study correlate symptoms of encephalitis with NMDA-R-Ab at early stages of the disease, including anxiety, agitation and seizures, although some of these symptoms re-emerge during the phase of recovery. Furthermore, the excitotoxicity caused by high levels of glutamate may account by the irreversibility of symptoms of some patients. Taken together with studies examining the cellular and synaptic effects of patients' antibodies [4], the current work reveals a novel mechanism of hyperglutamatergic state in the brain circuitry induced by antibodies.

### Summary on the effects of the glutamatergic synapse

**Figure 6:**
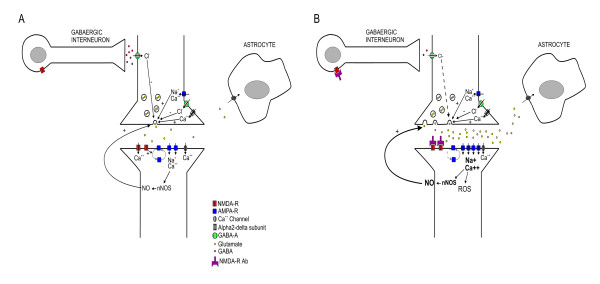
**Proposed scheme of the synaptic consequences of NMDA-R-Ab**. The antibodies block the NR1/NR2 heteromers of the NMDA receptor, causing a state of imbalance between NMDA and AMPA receptors. Blockade of NMDA-R impairs AMPA-R endocytosis. NMDA-R-Ab cause an increased release of glutamate which is dependent on the alpha2-delta subunit of VGCC and which is also related to a deregulation of gabaergic interneurons. NO acts as a retrograde messenger and amplifies glutamate release.

We suggest the following scheme of the effects of NMDA-R-Ab on glutamatergic synapses to summarize our findings (Figure 6). NMDA-R Ab block the NMDA-R not only at the post-synaptic level of the glutamatergic synapse, but also at the level of inhibitory gabaergic interneurons, a factor which contributes to the hyperglutamatergic state. The consequence of the fixation of NMDA-R-Ab to post-synaptic NMDA receptors is an imbalance NMDA/AMPA, rendering the post-synaptic element particularly vulnerable to administration of AMPA. Dysfunction of gabaergic interneurons results in a disinhibition of glutamatergic neurons, causing increased concentrations of glutamate and enhancing the excitability of neurons. The increase in NO concentrations participates in the glutamate release. Indeed, NO is a diffusible messenger which modulates synaptic transmission [23]. NO is known to act as a retrograde messenger and can amplify glutamate release [24].

### Potential implications

There are several potential clinical implications from this study. First, AMPA agonists might have deleterious consequences for patients. Second, the current results provide a rationale for evaluating treatments based upon AMPA antagonists in the acute phase of the disease. Third, the data robustly support an antibody-mediated pathogenesis of NMDA-R-Ab in patients' encephalitis [4,25], in agreement with clinical experience that shows that immunotherapy aimed to eliminate circulating antibodies is often effective. Overall, this study highlights that the encephalitis associated with NMDA-R antibodies is an auto-immune synaptic disorder.

## Abbreviations

NMDA: N-methyl-D-aspartate; AMPA: alpha-amino-3-hydroxy-5-methyl-4-isoxazole propionic acid; NO: nitric oxide NO; APV: 2-amino-5-phosphonovaleric acid; DNQX: 6,7-dinitro-quinoxaline-2,3-dione; PDC: L-2,4-trans-pyrrolidine-dicarboxylate; SNAP: S-nitroso-Nacetyl-D,L-penicillamine; 7-NI: 7-nitroindazole; BPU: blood per units.

## Competing interests

The authors declare that they have no competing interests.

## Authors' contributions

MM, JD, VR and JH contributed to the design of the experiments. JD, AD and JH were involved in the selection and follow-up of patients. MM and VR contributed to the experiments. All the authors have contributed to the interpretation of the results and have participated in the draft of the manuscript. All the authors have read and approved the manuscript.

## References

[B1] Dalmau J Gleichman AJ Hughes EG Rossi JE Peng X Lai M Dessain SK Rosenfeld MR Balice-Gordon R Lynch DR Anti-NMDA-receptor encephalitis: case series and analysis of the effects of antibodies Lancet Neurol 2008 7 1091 8 10.1016/S1474-4422(08)70224-2 18851928 PMC2607118

[B2] Dalmau J Tuzun E Wu HY Masjuan J Rossi JE Voloschin A Baehring JM Shimazaki H Koide R King D Mason W Sansing LH Dichter MA Rosenfeld MR Lynch DR Paraneoplastic anti-N-methyl-D-aspartate receptor encephalitis associated with ovarian teratoma Ann Neurol 2007 61 25 36 10.1002/ana.21050 17262855 PMC2430743

[B3] Seki M Suzuki S Iizuka T Shimizu T Nihei Y Suzuki N Dalmau J Neurological response to early removal of ovarian teratoma in anti-NMDA-R encephalitis J Neurol Neurosurg Psychiatry 2008 79 324 326 10.1136/jnnp.2007.136473 18032452 PMC2574536

[B4] Hughes EG Peng X Gleichman AJ Lai M Zhou L Tsou R Parsons TD Lynch DR Dalmau J Balice-Gordon RJ Cellular and synaptic mechanisms of anti-NMDA receptor encephalitis J Neurosci 2010 30 5866 75 10.1523/JNEUROSCI.0167-10.2010 20427647 PMC2868315

[B5] Manto M Laute MA Aguera M Rogemond V Pandolfo M Honnorat J Effects of anti-glutamic acid decarboxylase antibodies associated with neurological diseases Ann Neurol 2007 61 544 551 10.1002/ana.21123 17600364

[B6] Cais O Sedlacek M Horak M Dittert I Vyklicky L Jr Temperature dependence of NR1/NR2B NMDA receptor channels Neuroscience 2008 151 428 438 10.1016/j.neuroscience.2007.11.002 18068304

[B7] Manto M Laute MA A possible mechanism for the beneficial effect of ethanol in essential tremor Eur J Neurol 2008 15 697 705 10.1111/j.1468-1331.2008.02150.x 18445025

[B8] Manto M Laute MA Pandolfo M Depression of extra-cellular GABA and increase of NMDA-induced nitric oxide following acute intra-nuclear administration of alcohol in the cerebellar nuclei of the rat Cerebellum 2005 4 230 8 10.1080/14734220500243835 16321878

[B9] Corona JC Tapia R Calpain inhibition protects spinal motoneurons from the excitotoxic effects of AMPA in vivo Neurochem Res 2008 33 1428 34 10.1007/s11064-007-9559-7 18219574

[B10] Bergles DE Jahr CE Glial contribution to glutamate uptake at Schaffer collateral-commissural synapses in the hippocampus J Neurosci 1998 18 7709 16 9742141 10.1523/JNEUROSCI.18-19-07709.1998 PMC6792997

[B11] Gouix E Léveillé F Nicole O Melon C Had-Aissouni L Buisson A Reverse glial glutamate uptake triggers neuronal cell death through extrasynaptic NMDA receptor activation Mol Cell Neurosci 2009 40 463 73 10.1016/j.mcn.2009.01.002 19340933

[B12] Alvarez S Moldovan M Krarup C Acute energy restriction triggers Wallerian degeneration in mouse Exp Neurol 2008 212 166 78 10.1016/j.expneurol.2008.03.022 18486130

[B13] Tonnesen J Pryds A Larsen EH Paulson OB Hauerberg J Knudsen GM Laser Doppler flowmetry is valid for measurement of cerebral blood flow autoregulation lower limit in rats Exp Physiol 2005 90 349 55 10.1113/expphysiol.2004.029512 15653714

[B14] Bert L Favale D Jego G Greve P Guilloux JP Guiard BP Gardier AM Suaud-Chagny MF Lestage P Rapid and precise method to locate microdialysis probe implantation in the rodent brain J Neurosci Meth 2004 140 53 7 10.1016/j.jneumeth.2004.04.042 15589334

[B15] Wolf JA Moyer JT Lazarewicz MT Contreras D Benoit-Marand M O'Donnell P Finkel LH NMDA/AMPA ratio impacts state transitions and entrainment to oscillations in a computational model of the nucleus accumbens medium spiny projection neuron J Neurosci 2005 25 9080 9095 10.1523/JNEUROSCI.2220-05.2005 16207867 PMC6725747

[B16] Tapia R Medina-Ceja L Pena F On the relationship between extracellular glutamate, hyperexcitation and neurodegeneration in vivo Neurochem Int 1999 34 23 31 10.1016/S0197-0186(98)00061-8 10100193

[B17] Corona JC Tapia R AMPA receptor activation, but not the accumulation of endogeneous extracellular glutamate, induces paralysis and motor neuron death in rat spinal cord in vivo J Neurochem 2004 89 988 997 10.1111/j.1471-4159.2004.02383.x 15140197

[B18] Lau A Tymianski Glutamate receptors, neurotoxicity and neurodegeneration Eur J Physiol 2010 460 525 542 10.1007/s00424-010-0809-1 20229265

[B19] Norris CM Blalock EM Thibault O Brewer LD Clodfelter GV Porter NM Landfield PW Electrophysiological mechanisms of delayed excitotoxicity: positive feedback loop between NMDA receptor current and depolarization-mediated glutamate release J Neurophysiol 2006 96 2488 2500 10.1152/jn.00593.2005 16914613 PMC2756090

[B20] Marsden KC Beattie JB Friedenthal J Carroll RC NMDA receptor activation potentiates inhibitory transmission through GABA receptor-associated protein-dependent exocytosis of GABA(A) receptors J Neurosci 2007 27 14326 14337 10.1523/JNEUROSCI.4433-07.2007 18160640 PMC6673443

[B21] Iizuka T Yoshii S Kan S Hamada J Dalmau J Sakai F Mochizuki H Reversible brain atrophy in anti-NMDA receptor encephalitis: a long-term observational study J Neurol 2010 257 1686 91 10.1007/s00415-010-5604-6 20517615 PMC3071951

[B22] Sharma K Fong DK Craig AM Postsynaptic protein mobility in dendritic spines: long-term regulation by synaptic NMDA receptor activation Mol Cell Neurosci 2006 31 702 12 10.1016/j.mcn.2006.01.010 16504537

[B23] Kovacs R Rabanus A Otáhal J Patzak A Kardos J Albus K Heinemann U Kann O Endogenous nitric oxide is a key promoting factor for initiation of seizure-like events in hippocampal and entorhinal cortex slices J Neurosci 2009 29 8565 77 10.1523/JNEUROSCI.5698-08.2009 19571147 PMC6665664

[B24] Kano T Shimizu-Sasamata M Huang PL Moskowitz MA Lo EH Effects of nitric oxide synthase gene knockout on neurotransmitter release in vivo Neuroscience 1998 86 695 9 10.1016/S0306-4522(98)00179-1 9692709

[B25] Tüzün E Zhou L Baehring JM Bannykh S Rosenfeld MR Dalmau J Evidence for antibody-mediated pathogenesis in anti-NMDA-R encephalitis associated with ovarian teratoma Acta Neuropathol 2009 118 737 743 10.1007/s00401-009-0582-4 PMC2888642 19680671

